# Biological activities and therapeutic potential of soy isoflavones: a focus on anticancer activity

**DOI:** 10.1007/s11033-026-11922-8

**Published:** 2026-05-14

**Authors:** Maciej Kwieciński, Bartłomiej Kwiatkowski, Weronika Ziomek, Weronika Bomba, Paula Wróblewska-Łuczka

**Affiliations:** Department of Occupational Medicine, Department of Pathophysiology, Medical University of Lublin, ul. Jaczewskiego 8b, Lublin, 20-090 Poland

**Keywords:** Phytoestrogens, Soy isoflavones, Glycitein, Genistein, Equol, Cancer

## Abstract

**Abstract:**

Despite major advances in medicine, cancer treatment remains a considerable challenge. The rising incidence of new cases and the frequent resistance to cytostatics suggest that standard therapies may be insufficient. This highlights the need for novel compounds with potential anticancer activity and minimal side effects. Among natural molecules, soy isoflavones appear particularly promising. Soybeans and red clover are abundant in genistein and daidzein – isoflavones which are currently used as dietary supplements to alleviate menopausal symptoms, osteoporosis, and polycystic ovary syndrome. Reports also suggest potential benefits in neurodegenerative, cardiovascular, and metabolic diseases, including non-alcoholic fatty liver disease. Their mechanisms of action are diverse and extend beyond estrogenic activity, including anti-inflammatory, antioxidant, and immunomodulatory effects. In cancer, proposed mechanisms involve modulation of estrogen receptors, copper ion–dependent induction of cell death, promotion of apoptosis, inhibition of angiogenesis and metastasis, regulation of epigenetic processes, and effects on platelet function. Owing to their estrogen receptor interactions, isoflavones have been studied particularly in hormone-dependent cancers such as breast, ovarian, and prostate cancer. Moreover, in vitro and in vivo studies have reported promising results in other malignancies, including gliomas, neuroblastoma, hepatocellular carcinoma, lung and bladder cancers, osteosarcoma, and rhabdomyosarcoma. In summary, soy isoflavones require further investigation, particularly in combination with established chemotherapeutics, to evaluate their potential synergy with standard protocols. Their affordability, availability, and favorable safety profile strengthen their relevance as candidates for adjunctive cancer therapy.

**Graphical Abstract:**

**Figure:**
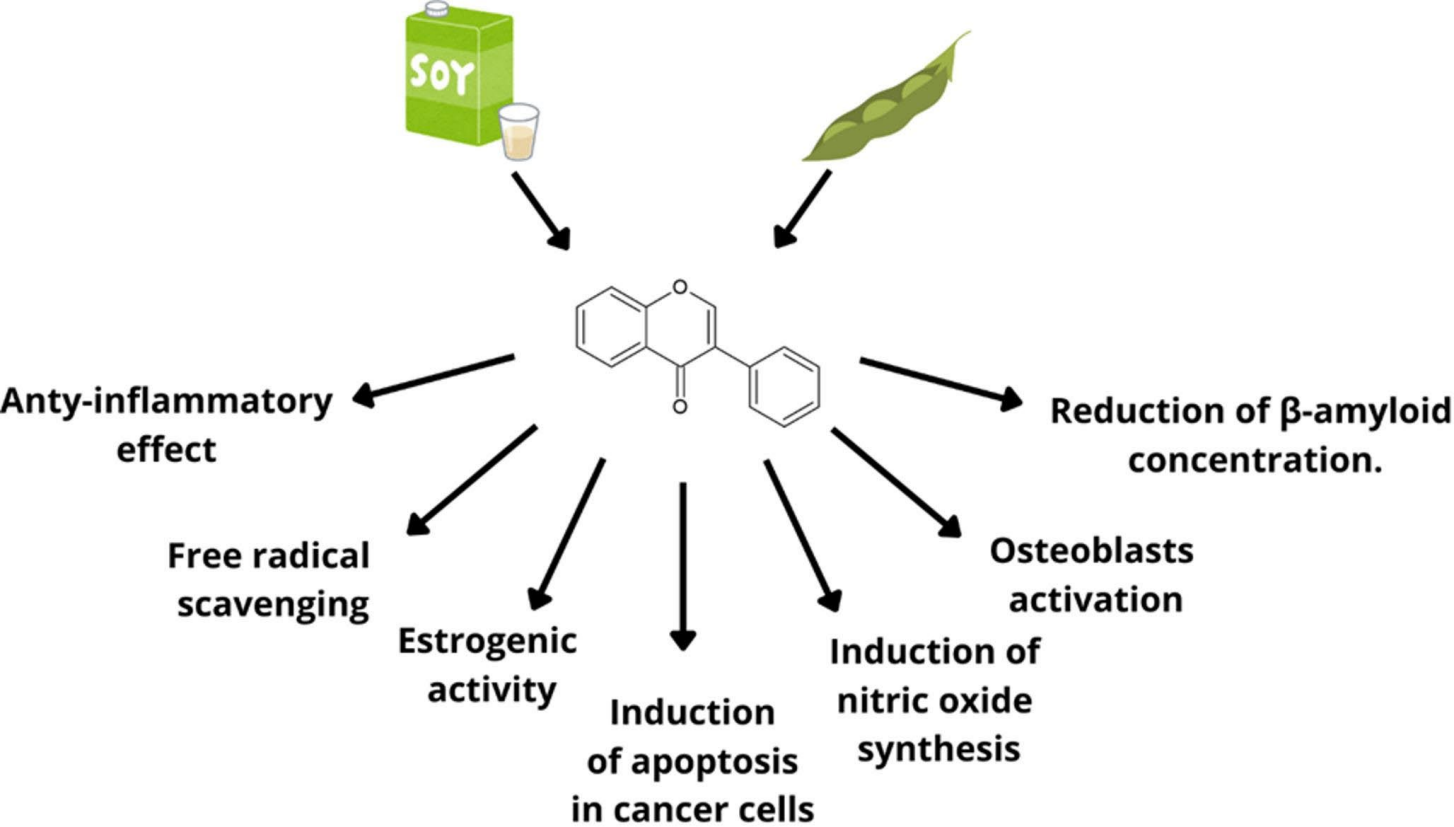
Pleiotropic mechanisms of action of soy isoflavones.

**Supplementary Information:**

The online version contains supplementary material available at 10.1007/s11033-026-11922-8.

## Introduction

Phytoestrogens, which have a hormonal action, have been recognised for many years, yet their pleiotropic effects on the body and new mechanisms of action are still being researched and discovered [1]. The most prevalent flavonoids detected in soy products are genistin, daidzin, and glycitein, i.e., genistein, glycitein, and daidzein, which are conjugated with glycosidic residues [1, 2]. The natural origin of these compounds and relatively widespread availability and low treatment costs are just some of the characteristics that enhance their possible use in supplementation in patients with a variety of indications [3]. Diseases in which soy isoflavones may be beneficial for human health include cardiovascular disease, polycystic ovarian syndrome, non-alcoholic fatty liver disease, obesity, osteoporosis, neurodegenerative diseases and menopause, but most importantly, cancers [1, 3–9]. However, this has not been clearly demonstrated in clinical trials, and some of the potential applications of isoflavones described in the ensuing article are based on in vitro observations. Furthermore, in the case of certain diseases, the results of clinical trials did not correspond with those obtained in vitro amd in some cases, they were even opposite [10, 11].

Isoflavones have attracted a considerable scientific interest in recent years, also due to their potential anticancer properties. According to data from epidemiological studies, people who consume soy have a lower risk of developing certain types of cancer, including cancers of the colon, breast and prostate, which are particularly common in the population [12]. The potential protective effect of a diet abundant in unprocessed plant products with a high fibre content is a plausible explanation, although the possibility of a beneficial additive effect of isoflavones cannot be discounted.

All of the above, inspired the authors to take up this topic. The aim of this review is to present the most important information on the therapeutic potential of soy isoflavones in the context of numerous diseases, with particular emphasis on their anticancer effects. The publication describes the effects of isoflavones in the context of many diseases as well the mechanisms of the potential anticancer effects of isoflavones, and summarises the available in vitro and in vivo research data, presenting the tested doses of compounds against individual types of cancer or specific cell lines.

## Literature review methodology

The authors conducted a comprehensive literature review using PubMed and Scopus databases, systematically filtering the available publications using the following search terms: ‘soy isoflavones’ ‘(isoflavones) AND (chemistry)’, ‘phytoestrogens’, ‘genistein’, ‘daidzein’, ‘glicytein’, ‘biochanin A’, ‘equol’, ‘metabolism’, ‘impact on human health’, ‘cancer’, ‘anti-cancer’ and ‘cell apoptosis’. The vast majority of the referenced articles were published between 2019 and 2024, however in order to examine the topic extensively, the scope was not limited to those years, encompassing older publications as well. The analysis included both original papers on clinical and pre-clinical studies, in addition to review papers and meta-analyses.

## Chemistry of isoflavones

**Fig. 1:**
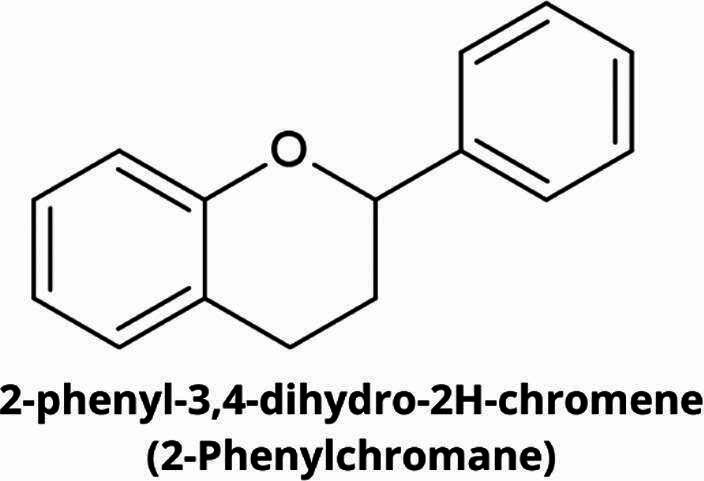
Flavan - the fundamental structural unit that is found in all flavonoids

**Fig. 2:**
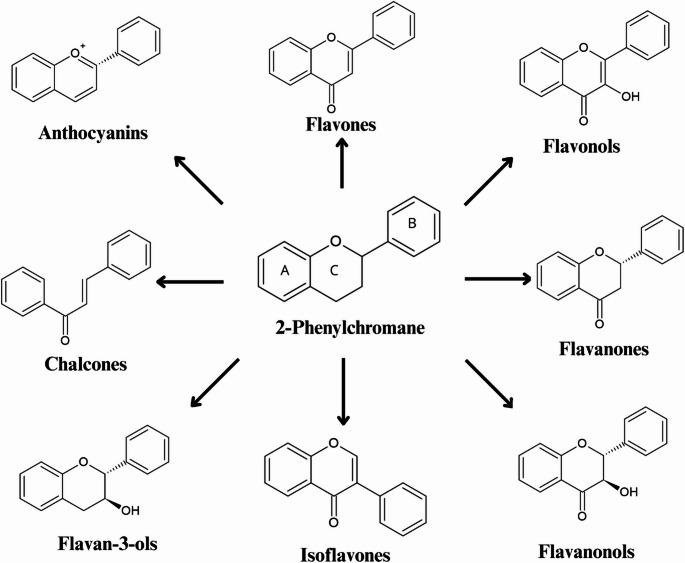
The most significant flavonoid subgroups

Flavonoids are a group of naturally occurring organic compounds that possess a 15-carbon skeletal structure. They are formed from two aromatic rings (A and B) and one heterocyclic ring [13]. The fundamental structure of flavonoids is thought to be 2-phenylchromane (2-phenyl-3,4-dihydro-*2 H*-1-benzopyran), commonly known as flavan (Fig. 1). The main differentiating factor between individual flavonoids is the presence of a double bond between C2 and C3, as well as the existence or absence of a carbonyl group at C4 in the C-ring. It is estimated that there are approximately 10,000 different types of flavonoids, which can be classified according to their chemical structures into several major groups, including flavones, flavonols, flavanones, isoflavones, flavan-3-ols (catechins), anthocyanins and chalcones (Fig. 2) [13].

All flavonoids are synthesised from the amino acid phenylalanine via the phenylpropanoid pathway. Phenylalanine is converted to *p*-coumaroyl-CoA by three enzymes: phenylalanine ammonia lyase (PAL), cinnamic acid 4-hydroxylase (C4H) and 4-coumarate : CoA ligase (4CL). Subsequently, *p*-coumaroyl-CoA and three molecules of malonyl-CoA are converted to chalcone by chalcone synthase (CHS), which represents a key reaction step that limits the rate of flavonoid synthesis. Then, chalcone isomerase (CHI) catalyses the intramolecular cyclization of chalcones to form flavanones, which results in the formation of the heterocyclic C-ring in the flavonoid pathway. Isoflavones are synthesised by the action of isoflavone synthase (IFS) on flavanones, forming intermediates that are then converted into genistein and daidzein with the participation of hydroxyisoflavanone dehydratase (HID). The action of IFS and HID results in the formation of a double bond between the C2 and C3 atoms of the C ring, as well as a shift of the B ring from the C2 position to the C3 position of the C ring. A variety of chemical modifications, including reduction, methylation, hydroxylation, glycosylation, and attachment of a malonyl group, result in the formation of the remaining isoflavones (Supplementary Materials, Fig. 3) [14].

Enzymes participating in the reactions are marked with abbreviations. C4H: Cinnamic acid 4-hydroxylase; CHI: Chalcone isomerase; CHS: Chalcone synthase; HID: Hydroxyisoflavanone dehydratase; PAL: Phenylalanine ammonia lyase.

The isoflavone synthesis pathway is of particular significance in a number of plant species, including soybean (*Glycine max*), red clover (*Trifolium pratense*), white clover (*Trifolium repens*), lucerne (*Medicago sativa*), peas (*Pisum sativum*), and green beans (*Phaseolus vulgaris*). The content of isoflavones in soybeans is 1.2–4.2 mg/g, whereas in red clover it is the highest and varies between 10 and 25 mg/g dry matter [1].

Isoflavones form a group of compounds listed in Supplementary Materials, Fig. 4. Of these, the most significant are the soy isoflavones, specifically daidzein, genistein, and glycitein, and those derived from red clover, including formononetin, biochanin A, daidzein, and genistein [1].

## Soy isoflavones

Soy isoflavones are the point of interest in numerous scientific studies due to their multidirectional and diverse effects on the human body. Among them, the following phytoestrogens are distinguished: genistein, daidzein, glycitein, biochanin A, and formononetin [1]. Equol is also an important compound exhibiting activity similar to the abovementioned phytoestrogens; however, as a metabolite of daidzein, formed by intestinal microbiota, it is classified as an isoflavan rather than an isoflavone and is not a plant-derived component [1, 7, 15].

## Metabolism of soy isoflavones

The main sources of soy isoflavones for humans are products made from soybean seeds and sprouts (1.5 mg/g) [1, 3, 16]. Minor amounts of them are also found in chickpeas and beans, fruits, vegetables and nuts, as well as milk from cows fed with red clover. The concentrations of isoflavones in cow’s milk vary depending on the cows’ feed composition. Available data indicate that 250 mL of cow’s milk contains approximately 0–28.1 µg of daidzein, 0–43.95 µg of genistein, and 0–47.28 µg of glycitein, while equol, a microbial metabolite of daidzein, may be present at concentrations ranging from 0.88 to 250.75 µg. In comparison, 250 mL of soya milk contains considerably higher amounts of total isoflavones (excluding equol), typically ranging from approximately 3.25 to 52.5 mg [1].

Soy isoflavones occur as aglycones (non-sugar components of glycosides) and glycosides [17]. Aglycones which represent less than 5% of the total isoflavones in soy are absorbed by passive diffusion in the small intestine, and their glucuronide metabolites are present in the peripheral blood afterwards [18]. However, the composition of soy products is dominated by isoflavones conjugated with carbohydrates, rather than in free form [19]. Indeed, isoflavones serve as signal molecules for the plant and must flow in the plant sap, As such they should be water-soluble and this is why they mainly occur in plants as glycosides [20]. β-glycosides, as hydrophilic and high molecular weight compounds, cannot be absorbed in the intestine and instead, they must be hydrolysed first [21]. This process occurs throughout the whole intestine and involves both human and bacterial enzymes. In the small intestine, specifically in the jejunum, glycosylated isoflavones are hydrolyzed at the enterocyte barrier by the human enzyme lactase-phlorizin hydrolase (LPH) [22]. Additionally, hydrolysis is performed by bacterial β-glucosidases, a process that becomes most intensive in the lower sections of the digestive tract [23]. The effect of these enzymatic reactions is the conversion of isoflavones into aglycones [24]. Genistein and daidzein, which are found in soybeans in the highest concentration, are further metabolised by UDP-glucuronyltransferase to β-glucuronides, and to a lesser extent by sulfotransferases to sulfate esters [1, 25]. Specifically, the conversion to sulfate occurs primarily when the glucuronosyl pathway becomes saturated. Therefore, the metabolic profile depends on the quantity of isoflavones ingested in a single meal. Subsequently, the abovementioned metabolites circulate in the blood plasma and are eventually excreted into the bile, deconjugated by intestinal bacteria and the lactase-phlorizin hydrolase, and reabsorbed, thereby participating in the enterohepatic circulation [1, 7, 16].

In addition to standard O-glycosides, it should be noted that isoflavones also form C-glycosides. The flagship example of these is puerarin, the 8-C-glucoside of daidzein, which is found in large quantities in the *Pueraria lobata* (kudzu) plant. There is a direct C-C bond in this compound between C-8 of daidzein and the carbohydrate group [26]. These compounds are relatively poorly absorbed in the gastrointestinal tract due to their high resistance to enzymatic hydrolysis compared to O-glycosyl-isoflavonoids [27]. While C-C bonds are highly resistant to standard enzymatic hydrolysis, recent studies have suggested the possibility of the hydrolysis of C-C bonds in puerarin by bacteria found in the human microbiome. In a particular study, two bacterial strains, *Lactococcus* sp. MRG-IFC-1 and *Enterococcus* sp. MRG-IFC-2, were isolated from the faecal samples of a healthy 43-year-old woman and were found to be capable of completely converting puerarin to daidzein [26]. This biotransformation is facilitated by a specialized dgp (C-deglycosylation of puerarin) gene cluster, which encodes enzymes that overcome the high stability of the C-glycosidic bond through a multi-step oxidative process [28].

Despite their poor absorption in the intestine, these compounds, as a result of their chemical structure, exhibit strong antioxidant properties, acting as free radical scavengers, particularly in the gastrointestinal tract, and contributing to the prevention of inflammatory and cancerous diseases [27, 29–31].

A variety of factors such as age, sex and daily food intake have been demonstrated to influence the bioavailability of soy isoflavones [23, 32]. In the context of age, it has been observed that levels of metabolites of soy isoflavones, in plasma and urine exhibit a notable age-related distinction during the initial months of life as compared to levels observed during adulthood [33]. Furthermore, a clinical study demonstrated that children (between 3 and 17 years of age) when given a weight-adjusted dose of soy nuts, showed a significantly higher urinary excretion rate for daidzein (+ 39%) and genistein (+ 44%) compared to adults [34].

In the context of women before and after menopause, studies have shown that age-related effects on isoflavone pharmacokinetics are compound-specific. While the bioavailability of genistein does not differ significantly between these two age groups, a statistically significant increase in the area under the concentration–time curve (AUC_0–t_) for daidzein has been observed in postmenopausal women. Despite this shift in total systemic exposure for daidzein, age appears to have no significant impact on other critical pharmacokinetic parameters for either daidzein or genistein, including half-life (T_1/2_), time to reach maximum serum concentration (T_max_), peak serum concentration (C_max_), apparent oral clearance (CL/F), or apparent volume of distribution (V_d_/F) [35]. However, it should be emphasized that in perimenopausal and postmenopausal women, despite comparable isoflavone bioavailability, the deficiency of endogenous estrogens may represent a critical factor modulating their overall biological efficacy [36]. In studies involving men and women who consumed various sources of soy isoflavones (soy milk, textured vegetable protein, and tempeh), it was observed that maximum serum daidzein concentrations were higher in premenopausal women, and that renal clearance of daidzein was lower compared with men after consumption of the same isoflavone dose, which may suggest higher bioavailability of soy isoflavones in premenopausal women than in men. However, no significant differences were observed between premenopausal women and men in the remaining pharmacokinetic parameters, including half-life T₁/₂, Tmax, AUC_0–t_, and volume of distribution normalized for bioavailability and body weight (Vd/(F·kg)) [35]. Similar results were observed in another study. During one month of soy milk consumption by women and men, women initially excreted higher amounts of both genistein and daidzein metabolites (24%, 66%) compared with men (15%, 47%). In addition, a longer excretion half-life of genistein was observed in women than in men after the first soy intake. Interestingly, over time, the excretion half-life of isoflavones gradually shortened in women but gradually lengthened in men [37]. These results suggest that the duration of soy consumption could also affect isoflavone bioavailability.

Moreover, ethnic differences have been demonstrated to influence soy isoflavone pharmacokinetics and bioavailability. Comparative studies reveal significant ethnic variations in the absorption of soy isoflavones, with individuals of Asian descent exhibiting higher bioavailability markers (C_max_ and AUC_0−t_) upon acute intake compared to Caucasians, regardless of the dietary context. Furthermore, chronic consumption leads to an increase in these markers only in the Caucasian population, highlighting ethnic origin as a additional factor of isoflavone systemic exposure [38].

In particular, the composition of the intestinal microbiome and the type of diet used are of significant importance [7, 32, 39–44]. For instance, an inulin-rich diet has been demonstrated to enhance the bioavailability of these compounds by directly stimulating the proliferation of beneficial intestinal microbiota, thereby increasing fermentation activity and biotransformation of isoflavones into active compounds [32]. As a result of the above factors, the bioavailability of isoflavones varies between individuals, but in general soy isoflavones are characterised by relatively high bioavailability in comparison to other biologically active compounds belonging to the flavone group [45]. A clinical study evaluating the bioavailability of isoflavones demonstrated that the total systemic exposure, expressed as mean AUC_0–t_, was significantly higher for daidzein (7978 ng·h/ml) compared to genistein (6150 ng·h/ml) following the administration of a daily dose in encapsulated form (a total of five capsules, each containing 17.85 mg of isoflavone glycosides). Concurrently, the T1/2 of genistein was found to be more than twice as long as that of daidzein, at 20.0 h and 9.71 h, respectively. These pharmacokinetic differences suggest that while daidzein predominates in plasma in terms of absolute quantity shortly after ingestion, genistein provides a more stable, long-term tissue exposure [46]. In premenopausal women following a vegetarian diet and consuming large amounts of soy, the total concentration of aglycone isoflavones in the blood was 729.60 ng·ml⁻¹, with genistein at 468.15 ng·ml⁻¹ (1.73 µM) and daidzein it was 253.17 ng·ml⁻¹ (0.99 µM). This suggests that a significant amount of the consumed isoflavones are absorbed and remain in circulation in an active form [47]. In women with high soy intake, circulating isoflavone concentrations may substantially exceed circulating estradiol concentrations, reaching levels reported to be up to 1,000–10,000-fold higher on a molar basis. Although most isoflavones circulate as conjugated, biologically less active forms, with aglycones representing only approximately 5–10% of the total pool, this may still be physiologically relevant, as endogenous estradiol is likewise largely protein-bound or present in conjugated forms. Thus, despite the relatively weak estrogenic potency of isoflavones, their much higher circulating concentrations may contribute to many of their observed in vivo and in vitro biological effects [48]. However, the ultimate physiological impact is not only determined by these concentrations, but also by the individual metabolic capacity to transform these compounds into more potent forms.

A crucial reaction, carried out by bacteria in the distal part of the small intestine and colon, is the conversion of daidzein to dihydrodaidzein (DHD) and ultimately to equol [7, 15]. Equol is more active, more easily absorbed and more stable than its precursor, making it a potent hormone compound [7, 19, 49]. Despite a marginally shorter half-life compared to daidzein (8.76 h vs. 9.34 h), this compound demonstrates a significantly lower plasma clearance (6.85 L/h vs. 17.5 L/h). Consequently, the predominant effect of reduced clearance ensures a higher degree of systemic exposure and enhanced bioavailability. In addition, it has been demonstrated to exhibit a heightened degree of binding affinity with oestrogen receptors [7, 39, 50]. The human intestinal microflora produces only S-equol enantiomers (Supplementary Materials, Fig. 5) [51]. Daidzein transformation reactions are specific only to certain groups of bacteria, examples of which are listed in Table 1.

**Table 1:** Equol and O-desmethylangolensin-producing bacteria in human microbiota

Substrate	Product	Bacteria classification	References
Dihydrodaidzein	Equol	*Adlercreutzia equolifaciens*	[7]
Dihydrodaidzein	Equol	*Eggerthella*	[7]
Daidzein	Dihydrodaidzein	*Clostridium*	[49]
Daidzein	Dihydrodaidzein	*Enterococcus*	[49]
Daidzein	Dihydrodaidzein	*Coprobacillus*	[49]
Daidzein	Dihydrodaidzein	*Pediococcus acidilactici*	[49]
Daidzein	Dihydrodaidzein	*Lactobacillus*	[7]
Daidzin	Daidzein	*Bifidobacterium*	[149]
Daidzein	Equol	*Bifidobacterium*	[149]
Daidzein	Equol	*Coriobacteriaceae*	[7]
Daidzein	Equol	*Pediococcus*	[49]
Daidzein	Equol	*Lactobacillus*	[7]
Daidzein	Equol	*Slackia*	[149]
Daidzein	Equol	*Adlercreutzia equolifaciens*	[49]
Daidzein	O-desmethylangolensin	*Eubacterium ramulus*	[53]
Daidzein	O-desmethylangolensin	*Clostridrium sp HGH 136*	[53]
Daidzein	O-desmethylangolensin	*Clostridium strain SY8519*	[53]
Puerarin	Daidzein	*Lactococcus sp. MRG-IFC-1* *Enterococcus sp. MRG-IFC-2*	[26]

Those bacterial species are present, according to various sources, from 30% of the population in Western Europe to 50% of the population in Asian countries [1, 7, 15]. People with this kind of gut microbiota have traditionally been classified in the scientific literature as ‘equol producers’ [7]. However, it is important to distinguish between the simple presence of equol-producing bacteria and the actual functional capacity to produce equol in physiologically significant amounts. Recent studies emphasize that not all individuals possessing the specific microbiota are classified as ‘equol producers’, as this status depends on specific diagnostic thresholds. Depending on the chosen cut-off values for equol concentrations (in urine or blood), the estimated prevalence of the equol-producer phenotype can vary significantly, ranging from 29% to nearly 48% within the same population [52]. The ability to produce equol is modulated by dietary patterns, specifically requiring a high intake of complex carbohydrates and fiber, while being inhibited by high-fat diets. This metabolic process is relatively stable in individuals, yet it remains highly susceptible to disruption by external factors such as antibiotic therapy, which can permanently impair the gut microbiome’s functional capacity to produce equolol regardless of dietary substrate availability [39]. Consequently, these individuals are particularly more predisposed to gain significant effects from the intake of soy products, than ‘equol non-producers’ [7]. Moreover, well-characterized equol-producing strains could be utilized as probiotics to enhance intestinal equol production, offering a potential strategy to optimize isoflavone metabolism in individuals with limited endogenous equol-producing capacity [7]. It should be noted, however, that isoflavone intake is not devoid of potential risks, similar to pharmacological interventions. Some evidence suggests they may function as endocrine disruptors, potentially interfering with thyroid homeostasis or promoting certain types of estrogen-sensitive tumours through their estrogenic activity [7]. Given these complexities and the inconsistent results reported across various human trials, the health impact of isoflavones appears to be highly dependent on individual physiological and microbial predispositions [7]. This may be the reason for the disparity in the examined effectiveness of intake of soy products on different individuals, as well as more favourable results in Asian populations compared to Europeans [1]. However, it is essential to note that these differences are not limited to equol production status alone. Recent studies demonstrate a significant inter-individual variability in isoflavone bioavailability. For instance, even under identical and chronic dosing conditions, plasma concentrations can differ significantly among subjects, remaining stable for a given individual but varies greatly between others [41]. Such variability suggests that the clinical response is modulated by the specific food matrix used and individual metabotypes—defined as groups of individuals sharing similar metabolic capacities, determined by an interaction of genetic polymorphisms, gut microbiota composition, age, and health status. This complex metabolic profile, rather than a universal dose-response relationship, may significantly influence the ultimate physiological impact of isoflavones [42, 43].

Another potentially significant intestinal metabolite of daidzein is O-desmethylangolensin (O-DMA). While O-DMA itself exhibits insignificant estrogenic potency, it serves as a key biomarker of specific intestinal microbiota activity. In contrast to equol, a diphenolic compound that is structurally analogous to 17β-estradiol, the C-ring in O-DMA was degraded by certain strains of intestinal bacteria. Therefore, by analogy, we can identify another specific population that is capable of producing O-DMA (O-DMA producers) [53]. Following soy consumption, approximately 10–40% of individuals do not excrete measurable levels of O-DMA in their urine, suggesting that the majority of people are “O-DMA producers”. Significantly, observational studies suggest that the absence of O-DMA-producing capacity (non-O-DMA producers) is associated with a less favorable breast cancer risk profile, lower bone density, and a higher prevalence of obesity. However, it seems that these associations are likely to reflect the broader metabolic activity of the bacterial ecosystem, rather than a direct physiological effect of O-DMA itself [40]. Other studies suggest that the ability to produce O-DMA and the ability to produce equol are independent of each other, i.e. having O-DMA-producing bacteria does not depend on having equol-producing bacteria. However, having both phenotypes in the same individual can affect O-DMA and equol concentrations in the blood or urine [53]. Bacterial strains that produce O-DMA from daidzein are shown in Table 1.

## Mechanism of isoflavone hormonal action

The hormonal action of soy isoflavones is by far the best known and clearly proven mechanism through which soy products can affect human health. However, women are particularly benefited in this aspect. Soy isoflavones are structural analogues of 17-β estradiol, which is why they are called phytoestrogens [54]. They have both weak agonist and antagonist activity, behaving much as a natural selective estrogen receptor modulator (SERM), depending on the current estrogen concentration and the distribution of the different types of estrogen receptors (ER) in the target organs [3, 55]. The antiestrogenic activity of isoflavones mainly involves functional antagonism against estradiol due to the competitive displacement of estradiol, which is 100–1,000 times stronger ligand. Thus, despite their agonistic activity, isoflavones may reduce the stimulation of estrogen receptors [56]. Hwang et al. studied the in vitro changes in gene transcription resulting from a decrease in oestrogen receptor activity, as observed at genistein, daidzein and equol concentrations above 10⁻⁷ M. These concentrations can be achieved in human blood serum through the consumption of soy products [56]. However, the above-mentioned phytoestrogen concentrations are maintained only for a short period when soy intake is not habitual. Therefore, only long-term consumption leading to stable plasma levels of isoflavones may exert anti-estrogenic effects.

Hypothesis suggests that soy isoflavones also affect the pituitary gland. This is supported by studies indicating their moderate effect on the hypothalamic-pituitary-gonadal axis, likely through changes in estrogen synthesis and steroid-metabolizing enzymes [57]. Watanabe et al. demonstrated that isoflavone supplementation in premenopausal women is associated with a lengthening of the menstrual cycle and subtle hormonal changes, suggesting that regular soy consumption may influence cycle length through neuroendocrine regulation other than direct estrogenic effects [58].

Individual phytoestrogens have different receptor affinity and potency from each other. Based on in vitro and in vivo studies, the following order was determined in terms of each phytoestrogen potency: estradiol > genistein and equol > glycitein > daidzein > formononetin and biochanin A [1]. The affinity of soy isoflavones to the β-estrogen receptor (β-ER) subtype is higher than for α-estrogen receptor (α-ER) [1, 5, 17, 32]. Those specifications have a significant impact on the hormonal activity of phytoestrogens toward different organs, according to the type of receptor predominant in them. β-ER receptors are predominant in the lungs, male reproductive organs, bladder, brain and skin, while α-ER are found mainly in the mammary gland, uterus, bones, pituitary gland and kidneys [44]. The effect on target organs has been observed primarily within in vitro studies. However, there is also an increasing number of in vivo studies on animal models, for example the impact of genistein on rat ovaries and clinical studies, such as examining the impact on human bone tissue [59–61].

Another type of oestrogen receptor that can potentially be activated by phytoestrogens is the non-classical G protein-coupled oestrogen receptor 1 (GPER1), which is involved in several biological processes. GPER1 is widely distributed and expressed in the skeletal muscle, neurons, vascular endothelium, various immune cells as well as in breast, ovarian, and lung cancer tissues.While its activity is not yet fully understood, it is known that GPER1 demonstrates a weaker binding affinity to oestrogens [44]. In vitro studies suggest that it may influence various signalling pathways [62]. Mostaphaoui et al. investigated whether soy isoflavones interact with GPER1. A triple-negative breast cancer cell line was treated with daidzein at doses of 25 µM, 50 µM and 100 µM for 12 and 24 h, which was shown to induce the expression of multiple GPER1 forms. Furthermore, GPER1 stimulation by daidzein was shown to lead to the activation of the downstream ERK and AKT pathways, suggesting a potential therapeutic application for this phytoestrogen in non-classical oestrogen signalling [63].

The estrogenic activity of isoflavones may also be attributable to their potential effect on low-capacity, high-affinity membrane estrogen receptors (mER) [64]. De Wilde et al. conducted an in vitro investigation with daidzein aglycones (from 10 pM to 1 µM) on osteoblasts isolated from parietal bones of 2-day-old female Wistar rats, which revealed that daidzein exhibits a binding affinity to the receptor in question, closely approximating the level exhibited by estradiol. This finding suggests the possibility that the isoflavone daidzein may utilise a similar membrane estrogen receptor (mER) to that employed by estradiol in order to induce its rapid effects [65]. Consequently, it can be hypothesised that soy isoflavones can bind to mER and produce rapid changes in signaling, similar to estradiol.

## Therapeutic potential of soy isoflavones

Soy isoflavones were originally used as dietary supplements to alleviate menopausal symptoms in women. The phytoestrogenic effects have attracted the attention of researchers, who have explored their potential use in the treatment of hormone-dependent cancers. This provided novel insights into the mechanisms of action of these compounds, which may find future application in various clinical conditions following further clinical evaluation.

The anticancer potential of soy isoflavones should be considered in the broader context of their multidirectional action and effects on the body. A wide range of effects observed in diseases not directly related to cancer, including osteoporosis, cardiovascular disease and metabolic disorders, may influence the overall efficacy and safety of their use. Analysis of these interactions allows for a more comprehensive assessment of the translational potential of isoflavones in the prevention and treatment of chronic diseases, including cancer.

### Menopause

Menopause is the final stage of ovarian physiology in women and represents the time when reproductive function is lost due to the complete depletion of the supply of ovarian follicles. It is characterised by fluctuations in reproductive hormone levels and changes in the menstrual cycle, causing such symptoms as irregular bleeding patterns, hot flashes, or vasomotor symptoms, vaginal dryness, dyspareunia, increased risk for urinary tract infections and even depression and anxiety [66, 67]. The symptoms listed above arise primarily from estrogen and progesterone deficiency, so the phytoestrogenic activity of isoflavones may have a beneficial effect in alleviating them. Since isoflavones have a higher affinity for the β-ER subtype, the main direct effect of genistein, daidzein, glycitein and equol is activation of β-ER, which essentially could help in restoring hormonal homeostasis [68]. Consequently, phytoestrogens have demonstrated favourable effects on the general well-being of postmenopausal women as well as reduction in vasomotor symptoms and hot flashes [69, 70]. Conversely, due to the lower expression of β-ER in the vaginal wall of postmenopausal in comparison to premenopausal women, isoflavones have not demonstrated a distinct beneficial effect on vaginal dryness or dyspareunia in various studies [67, 71–73]. It should be noted that in order to provide proper safety, all clinical studies on soy or isoflavone supplements were, and should be, conducted on women who had previously undergone bilateral mammography with no evidence of breast cancer.

When administered topically, positive effects on urogenital symptoms were demonstrated by isoflavone vaginal gel 4% and red clover (*Trifolium pratense*) vaginal cream 2% [67]. The effect was likely due to presence of biochanin A and formononetin activating β-ER, because in contrast to soybean, which contains mostly genistein and daidzein and their gycosides, red clover isoflavones are predominantly in methylated form [74]. In contrast, for oral delivery, specific effective doses for reducing menopausal symptoms have not yet been determined [68]. Several of the reviewed studies proved that consuming about 50 to 200 mg of soy isoflavones per day in the form of soy milk, tablets, capsules, powders etc. could help in reducing bothersome menopausal symptoms, such as hot flashes and urogenital symptoms [67, 68]. However, the results are not conclusive yet, because of the variability in the levels of estrogen during menopause and the differences in the bioavailability of isoflavones when administered in various forms of supplements in clinical studies.

However, according to various sources, the effects of excessive phytoestrogens can be harmful to women of reproductive age. Isoflavones can cause menstrual cycle disorders, endometriosis and even secondary infertility [75].

### Osteoporosis

Osteoporosis is a disorder of reduced bone mass and deterioration of its quality, affecting particularly elderly people and significantly increasing the risk of bone fracture. Since bone primarily contains ER-β, soy-derived products can be an effective alternative or addition to the standard hormone replacement therapy, because of their estrogen-like activity [6, 7, 76, 77]. They stimulate osteogenesis by promoting the expression of genes specific for osteoblast-activating factors [76]. In addition, soy isoflavones inhibit osteoclasts through activation of nuclear factor kappaB (NF-κB) [6, 76]. A recently identified mechanism through which isoflavones are likely to inhibit osteoporosis involves their capacity to stimulate angiogenesis. This process is mediated by the phosphoinositide 3-kinase/protein kinase B signaling pathway (PI3K/AKT) and epidermal growth factor receptor (EGFR) relay pathways, which prompt the migration of bone marrow endothelial cells (BMECs) and consequently stimulate the formation of H-type vessels within the cancellous bone, thereby enhancing osteogenesis [6]. The rapid, membrane estrogen receptor (mER)-mediated actions of isoflavones also appear to be biologically relevant for osteoblast function. In the aforementioned study by de Wilde et al., daidzein was shown to activate transcription factors regulating early-response genes involved in cell proliferation and differentiation, while also modulating the actin cytoskeleton, a key regulator of cell adhesion, division, and secretion [65]. The mechanisms previously described were studied in vitro on rats/mice osteoblast cell lines. The in vivo utilisation of animal models was instrumental in evaluating the comprehensive impact of the treatment on osteoporosis [6, 76, 78].

The potential of isoflavones in the treatment of osteoporosis is particularly promising, as evidenced by numerous clinical studies that have been collated in a meta-analysis. The evidence from 63 clinical studies indicates that isoflavone supplementation of either isoflavones or pure genistein compound, administered at a dose range of 4.5 to 600 mg/day or 30 to 54 mg/day, respectively. It was associated with significant increases in bone mineral density (BMD) at the lumbar spine, femoral neck, and distal radius in postmenopausal women. In addition, subgroup analyses revealed that beneficial effects on BMD were observed when the intervention duration was at least 12 months and when the daily genistein dose was 50 mg or higher [61].

### Polycystic ovarian syndrome

Polycystic ovarian syndrome (PCOS) is an endocrine and metabolic disorder occurring in about 6–20% of females in reproductive age [79]. The symptoms of PCOS involve serious reproductive dysfunctions, including infertility and pregnancy complications, unbalanced metabolic functions, such as insulin resistance, or type 2 diabetes, as well as psychological disorders, mainly depression and anxiety [80]. It has been suggested that genistein-containing isoflavone interventions may improve certain clinical manifestations of PCOS and be associated with favorable changes in ovarian morphology. Soy isoflavones affect the PCOS by reducing insulin resistance and regulating estrogen receptor activity, reducing oxidative stress and providing anti-inflammatory effects by many biological pathways [81–83]. Soy isoflavones have been shown to exert antioxidant effects in both experimental and clinical studies. For example, an in vivo study by Ma et al. demonstrated that oral administration of soy isoflavones (100 mg/kg) for 28 days in Sprague–Dawley rats with PCOS significantly improved estrous cycle regularity, reduced ovarian volume and weight, and attenuated oxidative stress and inflammatory markers, partly through suppression of NF-κB phosphorylation [81]. Consistent with these findings, Jamilian et al., in a randomized, double-blind, placebo-controlled trial involving 70 women aged 18–40 years with PCOS, reported that soy isoflavone supplementation significantly increased plasma total glutathione levels, an important antioxidant marker, while reducing malondialdehyde concentrations, indicating decreased oxidative stress [83].

### Neurodegenerative diseases

Preclinical evidence indicates that soy isoflavones exert neuroprotective effects through a complex interaction of multiple signaling pathways that extend beyond simple antioxidant activity. Recent comprehensive reviews emphasize their role in modulating key intracellular cascades, including the PI3K/Akt, MAPK/ERK, and Wnt/β-catenin pathways, which are essential for neuronal survival, synaptic plasticity, and brain development [84, 85]. Furthermore, isoflavones act as signal transducers by activating the Nrf2/ARE pathway, thereby enhancing the endogenous antioxidant defense system which is particularly relevant given their debated direct antioxidant capacity within the brain. A critical factor in this context is the role of equol, which exhibits significantly higher permeability across the blood-brain barrier (BBB) compared to its precursor, daidzein [84]. Recent studies in SH-SY5Y cells and in vivo models have demonstrated that equol exerts potent neuroprotective effects by effectively crossing the BBB and modulating oxidative stress and apoptotic pathways, suggesting that the neuroprotective potential of soy isoflavones is heavily dependent on individual microbial biotransformation [86, 87].

Furthermore, a crucial aspect of their mechanism is the interaction with the G protein-coupled estrogen receptor (GPER). Unlike classical nuclear receptors, GPER mediates rapid, non-genomic responses that protect neurons from excitotoxicity and neuroinflammation [84]. Importantly, the effects of isoflavones on cognition are highly complex and region-specific. Evidence suggests that their estrogenic signaling may exert different effects depending on the brain area, with potentially beneficial outcomes in the hippocampus and prefrontal cortex, while effects in other regions may vary or even be adverse depending on the developmental stage and hormonal environment [84].

These molecular mechanisms provide a broader context for results observed in specific animal models.

For instance, in female C57BL/6 mice, intraperitoneal administration of daidzein (25 mg/kg) was associated with an increased population of neuroprogenitor cells in the dentate gyrus of the hippocampus, as evidenced by markers such as doublecortin (DCX) [88]. While these experimental doses significantly exceed typical human dietary intake, they provide insight into the potential of daidzein to stimulate neurogenesis. Similarly, soy isoflavones have been shown to modulate oxidative stress parameters, such as increasing superoxide dismutase (SOD) activity and reducing lipid peroxidation markers (TBARS) in various mouse models [89, 90]. Research in Parkinson’s disease models has further demonstrated that daidzein and equol may alleviate mitochondrial dysfunction by activating the nuclear erythroid 2-related factor 2 (NRF2) pathway [91]. Furthermore, genistein has been shown to reduce β-amyloid concentrations in animal neural tissue, suggesting an anti-inflammatory effect [9].

While preclinical models provide essential mechanistic insights, the translation of these findings to human health remains a subject of ongoing debate, as clinical evidence is more limited and often inconsistent. A pilot clinical trial involving 24 patients with prodromal Alzheimer’s disease reported that 12 months of genistein administration led to a reduction in β-amyloid deposition and improved cognitive scores (MMSE, TAVEC, CDT) [92]. However, it must be noted that due to the small sample size and high inter-individual variability in isoflavone pharmacokinetics, these findings are preliminary and require confirmation in larger, well-powered trials. For instance, long-term supplementation with soy isoflavones in postmenopausal women did not show significant global cognitive benefits, although some domain-specific improvements such as in visual memory were [93]. Similarly, in patients with diagnosed Alzheimer’s disease, the administration of soy isoflavones failed to significantly enhance cognitive performance compared to placebo, with no significant differences in treatment effects observed between genders. However, exploratory analyses from these trials revealed that changes in specific cognitive domains were associated with plasma isoflavone levels; in particular, equol levels were positively correlated with speeded dexterity and verbal fluency [94]. This further supports the hypothesis that the clinical efficacy of soy-based interventions may depend on the individual’s ability to produce equol, rather than the total intake of parent isoflavones. While results from individual trials are often inconclusive, a comprehensive meta-analysis of randomized controlled trials indicates that soy isoflavone supplementation can significantly improve cognitive function, particularly in the domains of memory and executive function [95]. These findings suggest that the cognitive impact of isoflavones is domain-specific and may be more evident when analyzed across larger populations.

### Non-alcoholic fatty liver disease

Non-alcoholic fatty liver disease (NAFLD) is currently the most common chronic liver disease, with a reported prevalence of up to 25% of the global population [96]. The condition is characterised by the presence of hepatic steatosis, which may also be accompanied by other features of damage, provided that it is not attributable to alcohol consumption [96]. The prevailing hypothesis attributes this to a combination of the following factors: accumulation of lipids, oxidative stress, and lipotoxicity, which predominantly result from diabetes and obesity [96, 97]. Recent studies have revealed that the molecular mechanism of action of the components of soybean principally involves the modulation of three transcription factors, namely sterol regulatory element-binding protein-1, peroxisome proliferator-activated receptor-γ2 and fat-27-specific protein (lipid droplet-promoting protein), as well as the expression of their target genes involved in lipogenesis and lipolysis [97]. It can therefore be concluded that, as a result of their pleiotropic action, soy isoflavones could potentially have a beneficial effect in treatment and prevention of the aforementioned disorders [97, 98]. A number of clinical studies have demonstrated an inverse correlation between the intake of daidzein, genistein and glycitein and the occurrence of NAFLD and hyperlipidaemia. This association has been evidenced by improvements in clinical status and a significant reduction in ultrasonographically measured markers of hepatic steatosis, including the hepatic steatosis index (HSI), the fatty liver index (FLI) and the controlled attenuation parameter (CAP) [98]. However, it should be noted that the studies were conducted using soy products consumed by humans and not isoflavones alone [97]. Consequently, the findings cannot be considered fully conclusive yet, due to the potential beneficial effects of soy itself as a plant product with a low saturated fat content.

There is also evidence suggesting that the consumption of soy products may have a beneficial effect on alcohol-associated liver disease and chemical-induced liver damage. The hepatoprotective effect of isoflavones in these cases is assumed to be related to their antioxidant, anti-inflammatory, immunomodulatory and anti-fibrotic properties [99, 100]. This has been shown in both in vitro experiments, in which rat hepatocyte cell lines were exposed to genistein at concentrations of 5 µM and 10 µM, and in vivo experiments on rats administered intravenous solutions of isoflavones [101–103]. Furthermore, it is notable that soy isoflavones may possess the ability to restore autophagy activity during instances of chemical-induced liver disease [99]. Qin et al. observed that genistein, at concentrations of 10 µM and 25 µM, potently suppressed the ratio of p-Akt/AKT and p-mTOR/mTOR in HepG2 cells. This is likely due to the interaction of isoflavones with β-ER and the further inactivation of the Akt/mTOR pathway, which inhibits autophagy processes [104].

### Cardiovascular diseases

Cardiovascular diseases are the leading cause of mortality worldwide, with hypertension and atherosclerosis representing the most significant contributors to this burden [105].

Soy isoflavones, as well as equol, a metabolit of daidzein, have been suspected to have a beneficial effect on patients suffering from arterial and pulmonary hypertension [8, 106]. The ability of isoflavones to lower blood pressure has been documented in meta-analyses of a total of 32 studies in which patients were given soy isoflavone preparations for up to 12 weeks. The findings of these studies demonstrated a significant reduction in blood pressure [107, 108]. The antihypertensive effect of soy isoflavones is a result of their multidirectional action of isoflavones. Genistein has been shown to induce nitric oxide synthesis, increase cyclic adenosine monophosphate (cAMP) activity and inhibit tyrosine kinase, resulting in vasodilation, and lead to inhibition of the renin-angiotensin-aldosterone system (RAAS), affecting angiotensin-converting enzyme and angiotensin receptors activity, as well as lowering aldosterone levels [8]. Moreover, preclinical in vivo studies suggested, that soy isoflavones were capable of inhibiting hypoxia-induced hypertrophy of pulmonary artery smooth muscle cells, a crucial mechanism that leads to pulmonary hypertension [109, 110].

Due to their anti-atherosclerotic and elasticise effects, isoflavones may also be helpful in the prevention of cardiovascular disease (CVD) [1, 7]. Equol and other isoflavone metabolites, including O-DMA and 4-EP, have been shown to possess clinically significant anticoagulant activity [111]. The various metabolites affect platelets through different mechanisms. Equol most likely interferes with receptors for thromboxane [111, 112]. The 4-EP and O-DMA compounds, on the other hand, inhibit thromboxane synthase and influence directly calcium signalling, with 4-EP additionally inhibiting cyclooxygenase 1 [111, 112]. Meanwhile, although isolated isoflavone intake yields modest effects on lipids, whole soy protein products enriched with isoflavones have produced small but consistent reductions in low-density lipoprotein (LDL) cholesterol (~ 3%) in randomized trials [8]. Moreover, it has been suggested that many isoflavones possess antioxidative and anti-inflammatory effects, in addition to inducing nitric oxide production and could potentially help in maintaining a healthy endothelium and preventing endothelial cell dysfunction [8]. Kuriyama et al. investigated those mechanisms in in vivo study on rats with pulmonary hypertension and on human umbilical vein endothelial cells under a hypoxic environment exposed to genistein in concentrations from 10 µM to 30 µM [113]. However, up to date, results of various clinical studies suggest that soy isoflavones do not have an explicitly significant protective effect on cardiovascular risk [114]. It follows that further high-quality, randomized, controlled, large-scale studies are necessary.

The prevalence of obesity, along with its many attendant consequences, is also associated with increased cardiovascular risk. In a cross-sectional study involving 233 people, a statistically significant correlation was found between a higher incidence of obesity and the O-DMA non-producers phenotype. Such a correlation was not proven for equol non-producers [115].

The collective effect of these factors is the possible limitation of the development of atherosclerosis, hypertension and their potential consequences, cardiac or cerebral infarction, by the components of soybean, including soy isoflavones and their metabolites [8, 111].

### Anti-cancer properties

The treatment of many malignant tumours usually involves aggressive treatment, including the use of chemotherapy. To reduce the potential toxicity of the treatment, without losing its effectiveness, attempts are made to include naturally occurring, plant-based products as well. Soy isoflavones, especially genistein, demonstrate a spectrum of potentially beneficial effects in healthy cells distinct from their anti-cancer activity. At physiologically relevant concentrations (~ 0.5 µM), genistein activates estrogen receptors and downstream extracellular signal-Regulated Kinases 1 and 2 (ERK1/2) and NF‑κB signaling to upregulate antioxidant enzymes like manganese-SOD, thereby lowering baseline oxidative stress in non-malignant cells [116]. Animal models and clinical evidence also indicate that dietary soy isoflavones can reduce chronic inflammation, mitigating tumour necrosis factor α (TNF‑α)–mediated bone loss and vascular dysfunction. In bone tissue, isoflavones suppress receptor activator of nuclear factor κB ligand (RANKL) expression while increasing osteoprotegerin levels, potentially shifting the bone remodeling balance toward preservation and reducing resorption [117, 118]. Collectively, these findings highlight antioxidant, anti-inflammatory, and bone-preserving roles of soy isoflavones in healthy tissues, operating through mechanisms separate from their tumour-inhibitory pathways. However, it is important to note that many direct tumor-inhibitory effects of genistein reported in vitro have been observed at supraphysiological aglycone concentrations unlikely to be achieved in humans through habitual soy consumption, where total plasma isoflavone concentrations rarely exceed 5 µM and the free aglycone fraction typically constitutes ≤ 10% of the total.

Nevertheless, soy isoflavones may hold promise as adjuncts in cancer therapy by exerting additive effects or reducing treatment-related toxicity. Because many in vitro studies employ relatively high concentrations that may be difficult or undesirable to achieve systemically, particularly given potential adverse effects on healthy cells, the development of targeted delivery strategies capable of achieving therapeutic concentrations locally while minimizing systemic toxicity is of particular importance.

#### The importance of phytoestrogens affinity for the hormone receptor

Soy isoflavones, particularly genistein, exert biological effects that may be attributed to their molecular structure, which includes hydroxyl groups in positions analogous to those in estradiol. This structural resemblance underlies their preferential binding to ERβ, whereas endogenous estrogen binds with comparable affinity to both ERα and ERβ. The receptors differ mainly in recruited co-factors and transcriptional activity, which can be higher via ERα in several cell contexts [119]. Although the full implications of β-ER activation are not yet fully understood, current evidence suggests that genistein may modulate estrogen-dependent pathways, notably those involved in tumour growth and inflammation. Experimental studies have shown that genistein can downregulate genes associated with inflammatory responses in cancer cells, which may contribute to reduced tumour progression, although these effects can depend on the presence and interplay of estrogen receptors including ERα, ERβ, and GPER [119, 120]. Furthermore, a dual-phase response has been observed: low concentrations of genistein may stimulate cell proliferation, while higher concentrations tend to suppress growth and promote apoptosis [119]. These dose-dependent effects are particularly relevant in hormone-sensitive cancers such as breast and prostate malignancies. In men, some studies have suggested that soy isoflavones may be associated with modestly lower prostate specific antigen (PSA) levels, but the evidence is inconsistent and not conclusive. Preclinical (in vitro and animal) data on mechanisms such as decreased androgen receptor (AR) expression or attenuated prostate cancer cell growth often use high doses that may not translate to humans. Overall, the data remain mixed, with limited support from human studies for a protective effect against prostate cancer progression, and PSA is not considered an optimal biomarker for cancer outcomes [16].

#### Induction of cell death through a copper-dependent mechanism

Tumour transformation is accompanied by a dramatic increase in intracellular copper levels. Copper is required in tumour cells to increase endothelial cell proliferation and migration, and is also necessary for the release of angiogenic factors by tumour cells [12]. It is a metal ion present inside the nucleus, bound to deoxyribonucleic acid (DNA) bases, particularly guanine [121] Similar to tannic acid, curcumin, resveratrol and other plant polyphenols, flavonoids cause oxidative damage, inducing apoptosis in cancer cells, as demonstrated in various in vitro studies [12].

Previous studies have suggested that polyphenols, including isoflavones, may interact with intracellular copper and contribute to oxidative DNA damage in cancer cells, although these findings are largely based on in vitro models. There is a growing body of scientific research pointing to a mechanism of action of isoflavones specifically related to copper. The interactions between isoflavones and copper involve complex mechanisms of metal chelation and redox cycling that can modulate both the bioavailability of copper and the biological effects of isoflavones. Structurally, isoflavones bear specific functional groups that determine their capacity to chelate copper ions and to reduce cupric ions (Cu²⁺) to cuprous ions (Cu⁺), processes that are highly dependent on pH and molecular configuration. Chelation of copper ions by isoflavones occurs predominantly at the 5-hydroxy-4-keto moiety present in certain molecules such as genistein or biochanin A. However, these interactions have been primarily characterized under experimental conditions that may not fully reflect the in vivo environment. Chelation efficiency is influenced by pH, generally being more effective under neutral to slightly basic conditions. Moreover, the presence of a free 5-hydroxyl group, while essential for chelation, simultaneously diminishes the compound’s capacity to reduce copper ions by stabilizing the metal and thus limiting its accessibility for redox cycling.

Isoflavones also exhibit the capacity to reduce Cu²⁺ to Cu⁺, although the biological relevance of this process in vivo remains uncertain due to differences in achievable concentrations and physiological conditions. The copper-reducing ability depends strongly on the presence and position of hydroxyl groups on the isoflavone molecule. A free 4′-hydroxyl group on the B ring substantially enhances reduction potential, whereas replacement by methoxy groups attenuates this capacity. The 7-hydroxyl group of the A ring exerts minimal effect, though it may contribute to increased reduction at slightly alkaline pH (~ 7.5).

These chelation and reduction processes collectively influence isoflavones’ biological roles, notably their redox behavior, which can manifest as either antioxidant or prooxidant activities depending on concentration and context. At low to moderate concentrations, isoflavones typically exert antioxidant and anti-inflammatory effects by neutralizing ROS and protecting cells from oxidative stress. In contrast, at high concentrations or in specific cellular environments, isoflavones can act as pro-oxidants, i.e., induce the formation of reactive oxygen species. Such pro-oxidative activity can lead to the activation of defense mechanisms, including the NRF2 pathway – the master regulator of the oxidative stress response, thereby stimulating the antioxidant response [122]. Given that many of these effects have been demonstrated at supraphysiological concentrations, their direct relevance to in vivo conditions remains to be fully elucidated. The dual ability to both chelate copper and alter its oxidation state suggests that isoflavones can modulate copper bioavailability and facilitate or attenuate copper-mediated oxidative stress pathways [123].

Isoflavones, due to mobilisation of endogenous copper (potentially bound to chromatin) and interaction with it, can lead to production of ROS, initiate cellular DNA breakage, and induce apoptosis of cancer cells, as proposed in previous studies. This hypothesis is supported by in vitro evidence showing that isoflavones can reduce Cu²⁺ to Cu⁺ and mobilise copper, which may contribute to pro-oxidative effects in cancer cells with elevated copper levels. However, these metal-interaction properties were demonstrated at high concentrations (up to 5–10 mM) using mainly aglycone forms (and some glycosides) in cell-free systems. The study did not test circulating conjugated forms predominant in human plasma and lymph (such as glucuronides and sulfates), which are present at much lower concentrations in vivo. Therefore, the relevance of this copper-dependent mechanism to physiological conditions remains uncertain [121]. This hypothesis is further supported by experiments showing that the copper-specific chelator neocuproine significantly attenuated isoflavone-induced cytotoxicity and apoptosis, whereas iron (desferoxamine) and zinc (histidine) chelators had no effect. These findings were obtained in prostate cancer cell lines (LNCaP and DU145) at 50 µM isoflavone concentrations [12]. Collectively, these results suggest a selective role for copper in the pro-apoptotic effects of isoflavones under experimental conditions. Nevertheless, given that free aglycone forms occur at much lower concentrations in vivo, with most circulating as conjugated metabolites, the translational relevance of this copper-dependent mechanism for human anticancer activity remains unclear.

In addition, a study in normal MCF10A breast epithelial cells reported no detectable intracellular copper accumulation and, notably, no corresponding effect of soy isoflavones. These observations further support the hypothesis that at least some anticancer effects of isoflavones may depend on copper availability [12].

Another important aspect is that isoflavones reduce mRNA levels of the copper transporter 1 (CTR1) and copper-transporting ATPase 1 (ATP7A) proteins. The CTR1 protein controls the entry of copper into the cell by moving between the plasma membrane and intracellular vesicles. The ATP7A protein is responsible for importing copper into the Golgi apparatus and enabling the maturation of copper-dependent enzymes [124]. Concentrations of both of these proteins are higher in cancer cells. Farhan et al. studying prostate cancer cell lines revealed that mobilisation of endogenous copper resources and interference with the expression of two copper transporter genes (*CTR1* and *ATP7A*) in cancer cells, under the influence of consumed isoflavones, results in tumour growth suppression [12]. In the same study, the effect of isoflavones on copper metabolism is confirmed by the fact that reduced sensitivity of cells to the effects of genistein and daidzein was noted after attenuation of both copper transport proteins [12]. The concentrations of isoflavones involved in the study range between 10 and 50 µM. Achieving such a high concentrations of isoflavonoid in plasma in vivo is difficult or impossible in humans at safe doses.

#### Effects on cell division and apoptosis

As part of cancer tumour development, the rate of cell division increases. Anticancer compounds, i.e. genistein, can induce apoptosis - programmed cell death. This happens through interactions with specific receptors and signaling pathways, including binding to estrogen receptors (especially ERβ), inhibition of tyrosine kinase activity of EGFR and HER2 and activation of peroxisome proliferator-activated receptor γ (PPARγ), which collectively modulate pro-apoptotic signaling [5, 120]. The effect on the peroxisome proliferator-activated receptor (PPAR) is confirmed by in vitro studies assessing the effect of genistein on the proliferation of ELT-3 uterine leiomyoma cells induced by PPAR activation [125]. When soy isoflavones are used, an increase in PPAR expression, activation of caspase-3, decreased mRNA expression of protein phosphatase 2 A (CIP2A) as well as increased expression of DNA fragmentation are observed [120]. Activation of the breast cancer gene 1 (BRCA1) and ataxia telangiectasia and Rad3-related protein (ATR) complex, increases the DNA damage response, reduces *MET* protein expression, and stimulates the expression of microRNA-27a [5]. All these mechanisms are responsible for increasing the apoptosis of cancer cells, limiting tumour growth [5].

The cell division cycle is a set of events occurring in the cell’s organelles that lead to cell proliferation and duplication. Crucial mediators of this process are the enzymes that cut individual segments of the DNA chain and the kinases that transmit signals within the cells, inducing cell division. It has been shown in vitro that genistein at doses over 20 µM reduce breast cancer cell growth through estrogen receptor-independent inhibition of DNA topoisomerases and tyrosine kinases [54, 126]. Isoflavones exhibit significant differences in their biological activities due to variations in their chemical structures. Among these, genistein stands out for its unique ability to act as a specific inhibitor of tyrosine-specific protein kinases, which differentiates it from other isoflavones such as daidzein and glycitein. This kinase inhibition mechanism, observed with an IC50 of 20 µM, which allows genistein to interfere with key cellular signaling pathways involved in cancer cell proliferation and survival, including the EGFR, platelet-derived growth factor receptor (PDGFR), and downstream cascades like mitogen-activated protein kinase (MAPK) and PI3K/AKT. By competitively binding to the adenosine triphosphate (ATP) site of these kinases, genistein effectively induces cell cycle arrest and promotes apoptosis in tumour cells, which underlies its notable anticancer effects. Other isoflavones primarily exhibit phytoestrogenic or mild anti-inflammatory activities but lack the potent kinase-inhibitory actions of genistein. Therefore, understanding these distinctions is crucial for evaluating the therapeutic potential of individual isoflavones, particularly in the context of cancer prevention and treatment [127]. At the molecular level, genistein inhibits the growth of malignant cells by acting on a number of cell division cycle regulators and proteins, such as protein kinase B (AKT) and nuclear factor. This isoflavone reduces the activity of AKT, which regulates downstream signaling pathways including NF-κB. It has also been noted to be able to stop cell division in the G0/G1 phase, as well as to stop the G2/M phase by cyclin B [120].

#### Effect of genistein on angiogenesis and tumour metastasis

Genistein, by inhibiting the process of angiogenesis, when tested at doses over 20 µM, limits the availability of nutrients for cancer tissue, restricts its growth, and may even cause the reduction of its mass [3, 5]. Genistein and other isoflavones have antiangiogenic effects through the following mechanisms: inhibition of protein tyrosine kinase, suppression of vascular endothelial growth factor (VEGF) and basic fibroblast growth factor (bFGF), inhibition of matrix metalloproteinases (MMP-2 or MMP-9), suppression of cell adhesion and migration, inhibition of the JAK2/STAT3 pathway [128, 129]. This suggests a potential use a drug injected locally in a growing tumour.

Genistein has also been shown to be responsible for reducing the expression of matrix metalloproteinase (MMP) genes i.e. *MMP 2*,*3*,*15* when applied to T47D cells [120]. The AKT, hypoxia-inducible factor 1- α (HIF1-α) and VEGF cascades, also involved in vascular formation within the tumour when genistein is applied, are reduced. The use of soy isoflavones has been shown to inhibit angiogenesis, malignant cell migration, and reduce the invasiveness of cancer cells, making tumours less prone to metastasis [12, 130].

#### Regulation of epigenetic mechanisms

Genistein can alter key epigenetic modifiers. It acts as an inhibitor of DMTs or histone deacetylases (HDACs). Important genes often regulated by epigenetic mechanisms are *Cd74*, *Lpl*, *Ifi44*, *Sat1*, *Fzd9* and *Wwc1*. They are involved in the regulation of important signaling pathways during cancer development and progression. Another mechanism is the reversal of hypermethylation and restoration of PTEN expression in prostate cancer cells after treatment with genistein at a dose of 25 µM [131].

#### Effects of isoflavones on platelets

Soy isoflavonoids—most notably daidzein and genistein—may influence platelet behavior in ways that could impact cancer development and metastasis. For instance, daidzein at 12,5–50 µM (the use of such doses in vivo may be toxic to healthy cells) has been shown to inhibit platelet aggregation assessed by light transmission aggregometry triggered by collagen by reducing thromboxane A₂ synthesis, suppressing granule secretion, and modulating PI3K/AKT and MAPK signaling pathways while elevating intracellular cAMP levels. Since activated platelets are now recognized as key facilitators of tumour cell survival, immune evasion, and metastatic dissemination—including protection of circulating tumour cells (CTCs) and promotion of epithelial-to-mesenchymal transition (EMT) - this modulatory effect of isoflavonoids raises intriguing possibilities. By dampening platelet activation, isoflavonoids could reduce the ability of platelets to cloak CTCs, release growth factors, like tissue growth factor β (TGF-β) and VEGF, and enhance endothelial adhesion—thereby potentially disrupting critical steps in the metastatic cascade. While these interactions remain to be directly confirmed in cancer-specific in vivo models, the intersection between isoflavonoid-mediated platelet inhibition and platelet-driven cancer progression represents a promising avenue for future research [132, 133].

#### Soy isoflavones in combination with anticancer treatment

An interesting report is that genistein contributes to the potentiation of anticancer drugs [119]. It has been shown to enhance the effect of cisplatin on cervical cancer cells. The association of genistein and cisplatin produced the aforementioned effect by, among other things, increasing the levels of the tumour suppressor protein p53 in vitro [134]. Achieving such high concentrations of isoflavonoids in vivo is difficult, if not impossible, in humans at safe doses. A similar synergistic effect has been noted for the combination of genistein with centchroman for certain types of breast cancer cells [119]. The role of the compounds in question in combination with radiation therapy has been proven. Studies say that genistein or a mixture of isoflavones, contained in a traditional soybean diet, can be used to both potentiate the response of cancer cells to radiation therapy and reduce radiation-induced toxicity in normal tissues. Potentiation of the radiation effect of genistein observed in the range of 5–15 µM. genistein concentrations in this range are available doses obtained as a result of dietary supplementation [135]. In a study on PC-3 prostate cancer xenografts in mice, the use of genistein at a dose of 25 mg/kg/day with radiotherapy at 2 Gy/day was found to protect healthy tissues from radiation damage by inhibiting NF-kB, reducing inflammation and oxidative stress, which simultaneously increased the effectiveness of radiotherapy on cancer cells [136] .

In the context of cancer treatment, it is worth mentioning that soy isoflavones, especially daidzein, may have cardioprotective effects during chemotherapy [137].

#### Anticancer effects of soy isoflavones in in vitro studies

In vitro studies suggesting that isoflavones possess anticancer activity are available in the literature published to date. Many studies analysed the cytotoxic activity of soy isoflavones (pure substances or isolated extracts) [130]. The effect of the soy isoflavones were evaluated against tumour cell lines of glioblastoma multiforme, osteosarcoma and striated myxosarcoma. Another study confirmed the effect of genistein and daidzein at doses over 10 µM on apoptosis of prostate cancer cells [12]. An attempt was also made to evaluate the effect of genistein on breast cancer cells. This study showed that genistein reduces the expression of osteopontin (OPN), a protein that plays a key role in the progression and malignancy of breast cancer cells and the development of the tumour process, but also promotes the formation of distant metastases. Genistein led to a decrease in OPN secretion and thus inhibited the rate of formation, migration and invasion of cancer cells [138]. The summary of studies on the anticancer effects of soy isoflavones is presented in Table 2. The anticancer and cytotoxic effects of isoflavones, although evident in in vitro studies, require high concentrations, higher than those found in plasma, achieved through standard consumption of plant products or supplementation. The biological and therapeutic significance of such effects needs to be confirmed in in vivo models and at concentrations close to physiological levels.

**Table 2:** Anticancer effects of soy isoflavones (daidzein and genistein) on human cancer cell lines

Soy isoflavone tested	Type of cancer	Cell lines (human)	Tested doses [µM]	IC50	Effects	References
Daidzein+genistein (extract isolated from soybean molasses)	Glioblastoma multiforme	A-172, ATCC CRL-1620	61, 122, 245, 490, 980, 1960, 3900 µM	-	Dose-dependent cytotoxicity; significant reduction of cell viability and antioxidant activity vs. control	[130]
Daidzein+genistein (extract isolated from soybean molasses)	Osteosarcoma	Hos. ATCC CRL-1543	161, 122, 245, 490, 980, 1960, 3900 µM	-	Inhibited proliferation; decreased survival at ≥ 980 µM	[130]
Daidzein+genistein (extract isolated from soybean molasses)	Rhabdomyosarcoma	Rd. ATCC CRL-136	61, 122, 245, 490, 980, 1960, 3900 µM	-	Cytotoxic effect with concentration-dependent loss of viability	[130]
Genistein	Prostate cancer	LNCaP, DU145	10, 25, 50 µM	-	Induced apoptosis via copper-mediated oxidative stress; suppressed proliferation	[12]
Daidzein	Prostate cancer	LNCaP, DU145	10, 25, 50 µM	-	Reduced cell growth; promoted apoptotic cell death	[12]
Genistein	Prostate cancer	PC3, DU145	-	25 µM	Growth inhibition; cell-cycle arrest at G₂/M reported in PC3	[150]
Genistein	Breast cancer	MDA-MB-435 and MDA-MB-231	-	50 µM	Down-regulation of secreted osteopontin via MAPK/SIRT1; reduced metastatic potential	[138]
Genistein	Neuroblastoma	SK-N-SH	-	12.5 µM	Suppressed proliferation; promoted apoptosis through caspase activation	[5]
Genistein	Lung cancer	A549	10, 25, 50, 100, 200 µM	-	Reduced viability; induced G₂/M arrest and apoptotic markers	[5]
Genistein	Hepatocellular carcinoma	PLC/PRF5	1, 10, 25, 50, 75, 100 µM	-	Concentration-dependent inhibition of proliferation; promoted apoptosis	[5]

Abbreviations: IC50: Half maximal inhibitory concentration

#### Anticancer effects of soy isoflavones in in vivo studies

Results of in vivo studies available in the literature also support the anticancer properties of soy isoflavones. In one study, genistein was administered at 5 mg/kg bw/day to rats with fulminant liver failure, resulting in a significant reduction in functional impairment of the organ. It was shown that the action of genistein caused, among other things, a decrease in the expression of smooth muscle alpha-actin (α -SMA) and a decrease in the expression of the TGF-β. This factor affects epithelial-mesenchymal transition, which is closely related to the formation and distribution of metastatic tumour cells, implying that genistein may have a role in controlling the process of cancer metastasis [5]. In a study on bladder cancer, a reduction in final implanted tumour weight was observed in immune-depressed mice orally treated with genistein or soy phytochemical concentrate (SPC). It was associated with apoptosis of tumour cells and inhibition of angiogenesis within the tumour [5]. The results of the orthotopic bladder cancer model indicate that bioactive soy components (genistein, SPC) reach pharmacological concentrations in urine 1483–1559 µmol/L of free genistein. These levels are ~ 1000x higher than in blood (1.6 µmol/L) and 10x higher in orthotopic vs. subcutaneous tumours. The orthotopic model better reflects clinical conditions than the subcutaneous model [139]. Mice injected with human tumour cells for bone metastasis were then given genistein. A reduction in the number and volume of osteolytic metastases was observed [5]. The action of this isoflavone in the context of its effect on tumorigenesis is illustrated by a study using laying hens. These animals relatively often develop ovarian cancer. The hens were divided into 3 groups, fed different doses of genistein: control group − 3,01 mg/hen/dayno, low-dose genistein group − 52,48 mg/hen/day, high-dose group − 106,26 mg/hen/day. After a certain period of time and dissection of the animals, a significantly lower incidence of ovarian cancer was noted in hens fed low and high doses of genistein compared to the control group. The dose-dependent effect of isoflavone also proved to be significant - ovarian cancer was least likely to develop in the group fed a high dose of genistein. In addition, it was observed that the effect of genistein led not only to a reduction in the incidence of ovarian cancer, but also to a reduction in tumour size compared to the control group. Survival rates of hens were significantly higher in groups of animals fed with genistein than in groups that did not receive it [140]. In preclinical studies Chang et al. also proved that genistein can inactivate thyroid peroxidase in rats, counteracting the development of thyroid cancer without causing hypothyroidism [141]. There are clinical studies indicating a dose-dependent relationship between soy phytoestrogens and the progression of subclinical hypothyroidism. A dose of 16 mg/day increased the risk of progression (OR 3.6), especially with TSH > 6 mU/L, while a pharmacological dose of 66 mg/day did not show a significant increase in the incidence of overt hypothyroidism (above the expected spontaneous progression) [142, 143].

## Discussion and conclusions

The unique properties of soy isoflavones confirmed in many in vitro studies hold great promise for their possible future use in the treatment of many diseases including cancer. They are summarized and presented in Table 3.

**Table 3:** Impact of soy isoflavones on human health conditions and diseases

Health condition	Molecular mechanisms of action	Clinical effects	References
Menopause	Stimulation in β-ER activity	Restoration of hormonal balance; Reduction in the severity of menopause-related symptoms.	[54, 67, 68]
Osteoporosis	Promotion of the expression of genes specific to osteoblast-activating factors; Inhibition of osteoclasts by activation NF-κB; Angiogenesis stimulation by the by the PI3K/AKT and EGFR relay pathways	Stimulation of the osteogenesis, inhibition of osteolysis.	[6, 76]
Polycystic ovarian syndrome	Reduction of insulin resistance; Reduction of oxidative stress; Anti-inflammatory properties	Regression of the morphological changes in the ovaries; Reduction in the severity of PCOS symptoms	[81–83]
Neurodegenerative diseases	Oxidative stress inhibition (activation NRF2); Anti-inflammatory properties; Anti-apoptotic properties; Stimulation of the neurogenesis in the hippocampus; Reduction in the concentration of beta-amyloid in neural tissue	Improvement in cognitive function scores (MMSE, TAVEC, CDT)	[9, 85], 88– [92]
Non-alcoholic fatty liver disease	Modulation of the expression of sterol regulatory element-binding protein-1, peroxisome proliferator-activated receptor-γ2, lipid droplet-promoting protein and fat-27-specific protein	Reduction in lipid concentration; Reduction in ultrasonographically measured markers of hepatic steatosis (HSI, FLI, CAP)	[96–98]
Cardiovascular diseases	Induction of nitric oxide synthesis; Increasion of cAMP activity; Inhibition of tyrosine kinase; inhibition of the RAAS; Anti-oxidant properties; Anty-inflammatory properties	Anti-atherosclerotic effect; Elasticise effects; Prevention of endothelial cells dysfunction	[1, 7, 8, 109]
Cancer	Copper-dependent death; Induction of apoptosis; Phytoestrogenic affinity to tumours with estrogenic receptors; Inhibition of angiogenesis process related to MMPs and VEGF activity; Regulation of epigenetic mechanisms such as inhibition od DMTs and HDACs	Beneficial anti-cancer effects in in vitro and in vivo studies for cancers such as prostate, breast, ovarian, glioblastoma multiforme, osteosarcoma, myxosarcoma, bladder and thyroid cancers. Further research on humans, is required to evaluate the potential of soy isoflavones as adjuvants in cancer treatment.	[5, 12, 16, 54], 119– [121, 124, 126, 130, 150]

Abbreviations: β-ER: β-Estrogen receptor(s); NF-κB: Nuclear factor kappaB; PI3K/AKT: Phosphoinositide 3-kinase / Protein kinase B signaling pathway; EGFR: Epidermal growth factor receptor; PCOS: Polycystic ovarian syndrome; NRF2: Nuclear erythroid 2-related factor 2; MMSE: Mini-Mental State Examination, TAVEC: Complutense Verbal Learning Test; CDT: Clock Drawing Test; HSI: Hepatic steatosis index; FLI: Fatty liver index; CAP: Controlled attenuation parameter; cAMP: Cyclic adenosine monophosphate; RAAS: Renin-angiotensin-aldosterone system; MMP: Matrix metalloproteinase; VEGF: Vascular endothelial growth factor; DMTs: DNA methyltransferases; HDACs: Histone Deacetylases

So far, the observed estrogenic and cytotoxic activity at dietary doses, induction of cell apoptosis and antiproliferative activity observed at pharmacological doses are among the most important mechanisms by which isoflavones, such as genistein and daidzein, affect cancerogenesis. It is important to expand research and conduct further studies on the molecular mechanisms of action of these compounds, so that they can be used effectively in the treatment of patients beyond oncology. They can bring great value in personalised treatment by local injections in the tumours.

So far, the observed antiproliferative activity (cell-cycle arrest) and cytotoxic activity (apoptosis induction and cell death) are among the most important, though related, mechanisms by which isoflavones such as genistein and daidzein influence carcinogenesis. Clarifying how these dose- and context-dependent effects arise at the molecular level is essential, so that these compounds can be used effectively in the treatment of patients beyond oncology. They may bring significant value in personalised treatment. Therapies whose efficacy has not yet been established, such as oral administration of soy isoflavones in the treatment of malignant neoplasms in humans, cannot ethically be considered as primary treatment modalities for severe diseases. Accordingly, the models described herein are limited to illustrating potential mechanisms that may support cancer therapy and warrant further investigation.

Nevertheless, the precise doses required to optimise the effectiveness of dietary soy isoflavone supplementation in humans remain unreliable. This is primarily attributable to differences in the metabolism of these compounds across diverse populations, along with the presence of ‘equol producers’ and ‘equol non-producers’. In individuals who are ‘equol non-producers’, the efficacy of phytoestrogens is significantly reduced due to not having an adequate intestinal flora. This phenomenon may be the reason why the research performed by Fan et al. revealed that a diet comprising of higher quantities of soy-derived products has been shown to be associated with a significant (approximately 10%) reduction in cancer risk, while it did not reach a statistically important impact on cancer mortality [4]. Moreover, the absence of standardised dosage methods for soy-derived products supplementation poses a significant challenge in conducting objective comparisons of study results from different research centres. Further research on soy isoflavones’ impact on human health is necessary and recommended, especially in clinical conditions, due to the promising results of already conducted studies, presented in this review.

In the context of an ageing population and an increasing annual prevalence of the diseases discussed in this article, nutritional interventions and the promotion of awareness regarding healthy lifestyles appear to be of particular significance in the prevention of the aforementioned diseases. Isoflavone-rich foods, including soybeans, can be regarded as a valuable nutraceutical foodstuff due to their high concentration of bioactive compounds. The term “nutraceutical” is generally understood to mean a purified product derived from a human food source that is intended to provide additional health benefits beyond the basic nutritional value found in food [144]. Isoflavone-rich foods, such as soybeans, can be regarded as a valuable nutraceutical foodstuff due to their abundant estrogenic compounds. However, it should be noted that, despite the growing body of scientific evidence in favour of the beneficial effects of soy isoflavones, further randomised studies are still required to assess the actual therapeutic potential of these substances on a large group of patients.

Furthermore, despite the existence of strong concerns postulating that the consumption of soy products rich in estrogenic compounds is associated with the feminization of males, meta-analyses and clinical data demonstrate that moderate soy consumption is not associated with a reduction in male sex hormone concentrations nor with a deterioration in semen quality [145, 146]. There are a few case studies in the literature which suggest that consuming large quantities of soy milk (an average of 300 mg of isoflavones per day, equivalent to 0.5 to 1.2 L of soy milk) over a period of over two years can lead to erectile dysfunction, decreased androgen levels and gynecomastia [147, 148]. Nevertheless, these reports concern cases of extremely high isoflavone consumption and are not clinically significant in relation to the aforementioned meta-analyses.

## Electronic Supplementary Material

Below is the link to the electronic supplementary material.

Supplementary Material 1

## Data Availability

No datasets were generated or analysed during the current study.

## Abbreviations

4-EP: 4-ethylphenol
4CL: 4-coumarate: CoA ligase
6-OHDA: 6-hydroxydopamine
α-ER: α-Estrogen receptor(s)
α-SMA: α-Smooth muscle actin
AKT: Protein kinase B
AR: Androgen receptor
ATP: Adenosine triphosphate
ATP7A: Copper-transporting ATPase 1
ATR: Ataxia telangiectasia and Rad3-related protein
AUC_0–t_: Area under the concentration–time curve
β-ER: β-Estrogen receptor(s)
bFGF: Basic fibroblast growth factor
BMD: Bone mineral density
BMECs: Bone marrow endothelial cells
BRCA1: Breast cancer gene 1
C4H: Cinnamic acid 4-hydroxylase
cAMP: Cyclic adenosine monophosphate
CAP: Controlled attenuation parameter
CDT: Clock Drawing Test
CHI: Chalcone isomerase
CHS: Chalcone synthase
CIP2A: Cancerous inhibitor of protein phosphatase 2 A
CL/F: Apparent oral clearance
C_max_: Peak serum concentration
V_d_/F: Apparent volume of distribution
CTCs: Circulating tumour cells
CTR1: Copper transporter 1
CVD: Cardiovascular disease
DCX: Doublecortin
DHD: Dihydrodaidzein
DMTs: DNA methyltransferases
DNA: Deoxyribonucleic acid
EGFR: Epidermal growth factor receptor
EMT: Epithelial-to-mesenchymal transition
ER: Estrogen receptor(s)
ERK1/2: Extracellular signal-Regulated Kinases 1 and 2
FLI: Fatty liver index
GPER1: G protein-coupled estrogen receptor 1
HDACs: Histone Deacetylases
HID: Hydroxyisoflavanone dehydratase
HIF1-α: Hypoxia-inducible factor 1-α
HSI: Hepatic steatosis index
HRT: Hormone replacement therapy
IC50: Half maximal inhibitory concentration
IFS: Isoflavone synthase
LDL: Low-density lipoprotein
MAPK: Mitogen-activated protein kinase
mER: Membrane estrogen receptor
MMP: Matrix metalloproteinase
MMSE: Mini-Mental State Examination
NAFLD: Non-alcoholic fatty liver disease
NF-κB: Nuclear factor kappaB
NRF2: Nuclear erythroid 2-related factor 2
O-DMA: O-desmethylangolensin
OPN: Osteopontin
PAL: Phenylalanine ammonia lyase
PCOS: Polycystic ovarian syndrome
PDGFR: Platelet-derived growth factor receptor
PDX: Patient-derived xenografts
PI3K: Phosphoinositide 3-kinase
PI3K/AKT: Phosphoinositide 3-kinase / Protein kinase B signaling pathway
PPAR: Peroxisome proliferator-activated receptor
PSA: Prostate-specific antigen
RAAS: Renin-angiotensin-aldosterone system
RANKL: Receptor activator of muclear factor κB ligand
ROS: Reactive oxygen species
SERM: Selective estrogen receptor modulator
SOD: Superoxide dismutase
SPC: Soy phytochemical concentrate
T_1/2_: Half-life
TAVEC: Complutense Verbal Learning Test
TBARS: Thiobarbituric acid reactive substances
TGF-β: Tissue growth factorβ
T_max_: Time to reach maximum serum concentration
TNF‑α: Tumour necrosis factorα
T-AOC: Total antioxidant capacity
Vd/(F·kg): Volume of distribution normalized for bioavailability and body weight
VEGF: Vascular endothelial growth factor
