# Supplementary material for: Biological activities and therapeutic potential of soy isoflavones: a focus on anticancer activity
Source: Mol Biol Rep. 2026 May 14;53(1):769. doi: 10.1007/s11033-026-11922-8 (PMC13176004; doi:10.1007/s11033-026-11922-8)
Supplement: Supplementary file 1 — Supplementary Material 1 [file 11033_2026_11922_MOESM1_ESM.docx]

**Supplementary Materials**

**Biological activities and therapeutic potential of soy isoflavones: A focus on anticancer activity.**

Maciej Kwieciński (M.K.)^1^, Bartłomiej Kwiatkowski (B.K.)^1^, Weronika Ziomek (W.Z.)^1^, Weronika Bomba (W.B.)^1^, Paula Wróblewska-Łuczka (P.W-Ł.)^1^

^1^Department of Occupational Medicine, Department of Pathophysiology, Medical University of Lublin, ul. Jaczewskiego 8b, 20-090 Lublin, Poland;

Maciej Kwieciński^1^, [m.kwiecinski.med@gmail.com](mailto:m.kwiecinski.med@gmail.com), <https://orcid.org/0009-0006-1055-8763>

Bartłomiej Kwiatkowski^1^, [bartekchemlek@gmail.com](mailto:bartekchemlek@gmail.com), <https://orcid.org/0009-0006-0472-9095>

Weronika Ziomek^1^, [weronikaziomek9@gmail.com](mailto:weronikaziomek9@gmail.com), <https://orcid.org/0000-0002-8788-5299>

Weronika Bomba^1^, [weronikabomba8@gmail.com](mailto:weronikabomba8@gmail.com), <https://orcid.org/0009-0004-8481-3603>

Paula Wróblewska-Łuczka^1^, [paula.wroblewska-luczka@umlub.pl](mailto:paula.wroblewska-luczka@umlub.pl), <https://orcid.org/0000-0002-7099-1968>

Corresponding author: Paula Wróblewska-Łuczka, paula.wroblewska-luczka@umlub.pl; Phone: +48-81-448-6508, Fax: +48-81-448-6501

The running head: ‘Anticancer potential of soy isoflavones’


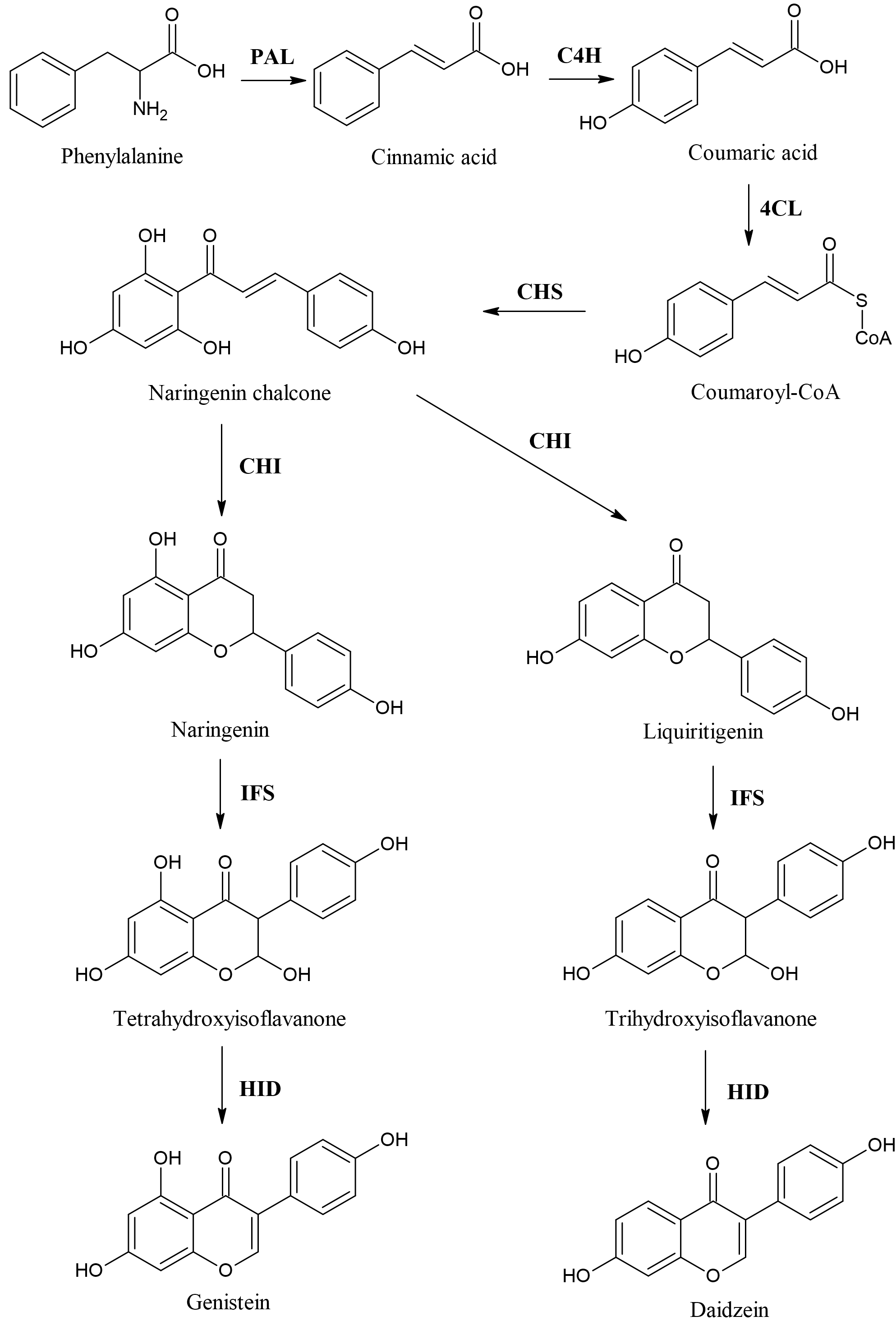


Figure 3. The pathway of soy isoflavone synthesis from phenylalanine.


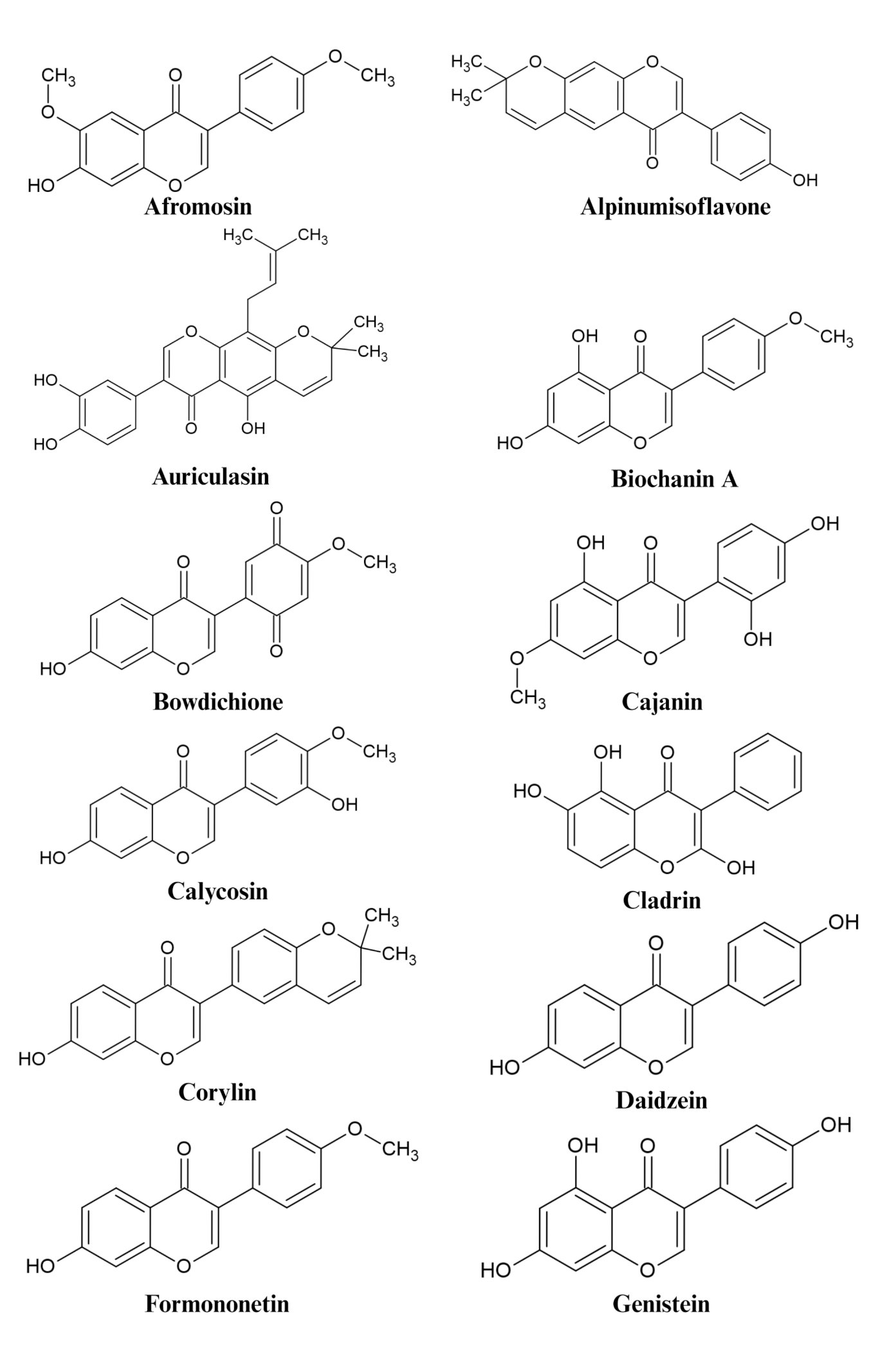

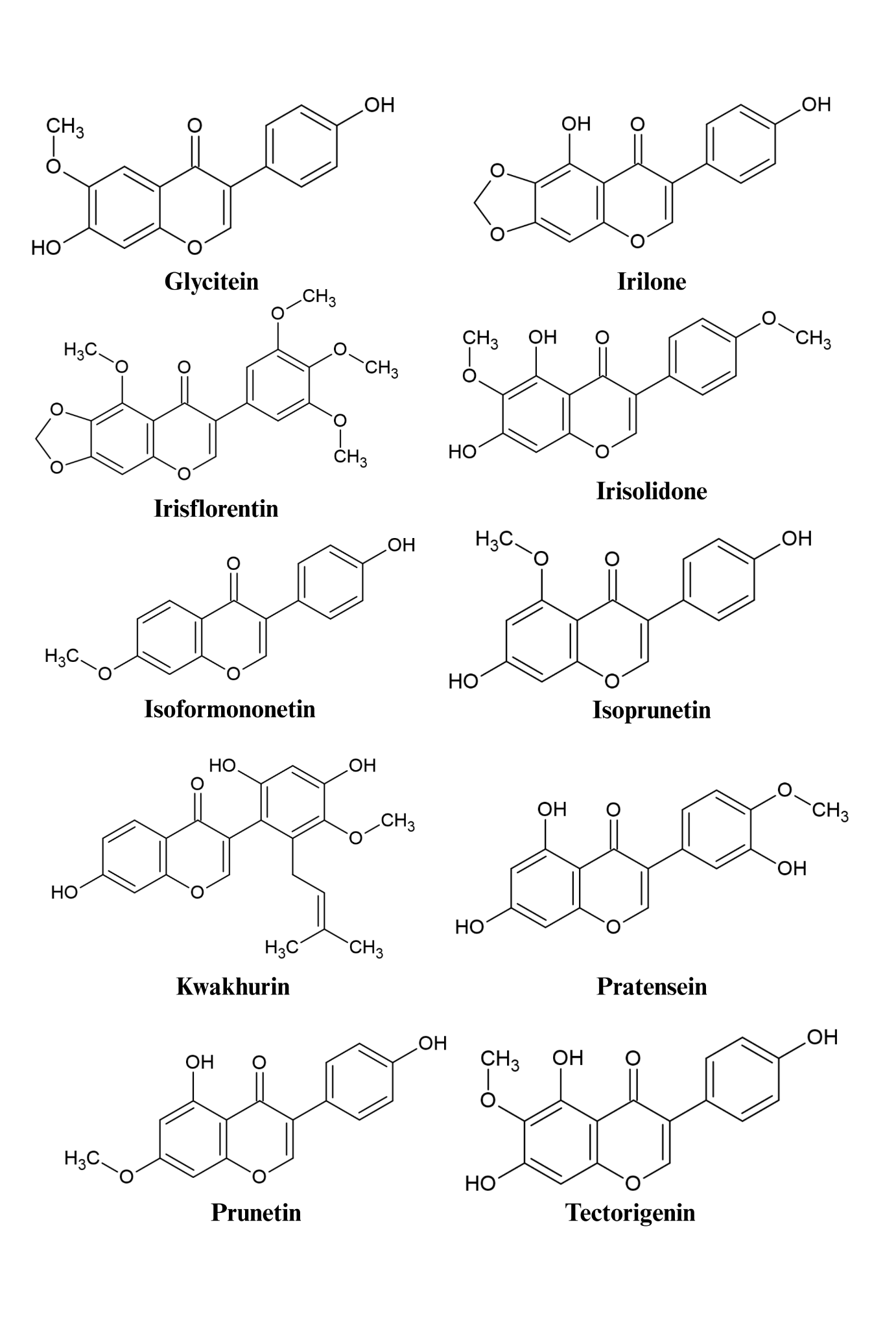


Figure 4. Chemical structures of the most important isoflavones.


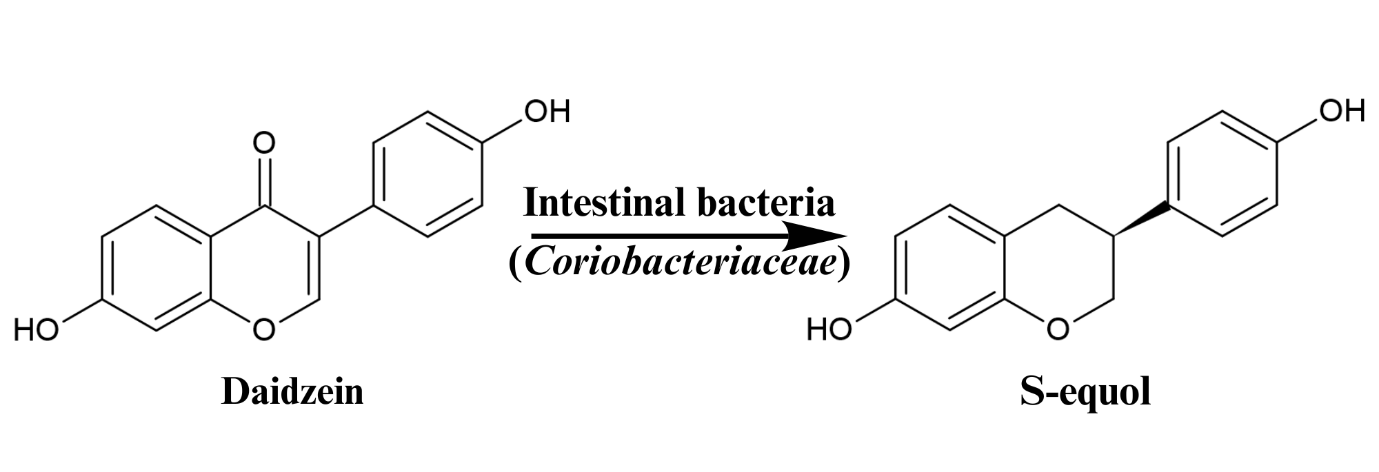


Figure 5. The biotransformation reaction of daidzein to S-equol by gut bacteria
